# Biomarker-Based Targeting of the Androgen-Androgen Receptor Axis in Advanced Prostate Cancer

**DOI:** 10.1155/2012/781459

**Published:** 2012-08-22

**Authors:** Manish Kohli, Rui Qin, Rafael Jimenez, Scott M. Dehm

**Affiliations:** ^1^Department of Oncology, Mayo Clinic, 200 First Street SW, Rochester, MN 55905, USA; ^2^Department of Health Sciences Research, Mayo Clinic, 200 First Street SW, Rochester, MN 55905, USA; ^3^Department of Laboratory Medicine and Pathology, Mayo Clinic, 200 First Street SW, Rochester, MN 55905, USA; ^4^Masonic Cancer Center and Department of Laboratory Medicine and Pathology, University of Minnesota, Minneapolis, MN 55455, USA

## Abstract

Recent therapeutic advances for managing advanced prostate cancer include the successful targeting of the androgen-AR axis with several new drugs in castrate resistant prostate cancer including abiraterone acetate and enzalutamide (MDV3100). This translational progress from “bench to bed-side” has resulted in an enlarging repertoire of novel and traditional drug choices now available for use in advanced prostate cancer therapeutics, which has had a positive clinical impact in prolonging longevity and quality of life of advanced prostate cancer patients. In order to further the clinical utility of these drugs, development of predictive biomarkers guiding individual therapeutic choices remains an ongoing challenge. This paper will summarize the potential in developing predictive biomarkers based on the pathophysiology of the androgen-AR axis in tumor tissue from patients with advanced prostate cancer as well as inherited variation in the patient's genome. Specific examples of rational clinical trial designs incorporating potential predictive biomarkers from these pathways will illustrate several aspects of pharmacogenetic and pharmacogenomic predictive biomarker development in advanced prostate cancer therapeutics.

## 1. Introduction

Prostate cancer (PCa) is the second leading cause of cancer-related mortality in US men with an estimated 33,720 deaths in 2011 [1]. Virtually all PCa-related deaths occur in patients with metastatic-stage disease, the initial treatment for which is androgen deprivation therapy (ADT) [2, 3]. In addition to advanced metastatic stage disease, ADT has also been used for treating locally advanced PCa and for biochemically relapsed disease after failure of localized-stage treatments with radical prostatectomy or radiation therapy. It has been estimated that a third of the over 2.3 million men with PCa in the US received ADT in 2007 as part of their care [4]. ADT therefore constitutes a significant clinical therapy for PCa patients. However, while it provides effective control of disease for variable time periods [5–8] in metastatic PCa patients, ADT also contributes to side effects including osteoporosis, loss of sexual libido, increased risk of diabetes and coronary artery disease, and metabolic syndrome [9]. Several challenges therefore remain in the use of ADT in PCa. Foremost is the lack of validated biomarkers predictive of treatment response to ADT or side effects of ADT which can be incorporated into designing clinical trials that optimize ADT treatment effects. Since the physiological basis of ADT action is to block the production or action of androgens, several aspects of androgen-androgen receptor (AR) axis function can potentially form critical elements in developing prognostic and predictive biomarkers of ADT response and toxicity. Scientific enquiry into the development and application of tumor markers is proceeding rapidly in all tumor types. However, in advanced PCa, this explosion in biomarker research interest unfortunately has not always translated into design of studies to formally assess the value of biomarkers in clinical practice. Furthermore, at an even more basic level, the steps necessary to develop prognostic and predictive biomarkers in PCa from an interesting laboratory observation to a clinically valuable and validated tool for improving the treatment of patients with advanced cancer have not been well defined. 

This paper will evaluate potential opportunities for androgen-AR axis-based biomarker development with a specific focus on somatic genomic alterations of the AR and components of the androgen-AR axis. Emerging evidence of germline variation in androgen-AR axis genes and their effects on clinical outcomes of ADT responses in advanced PCa will also be discussed. Finally, the paper will present potential clinical design models and scenarios that incorporate androgen-AR axis-based biomarkers into the design of PCa therapeutic trials that use novel and emerging agents targeting androgen-AR axis biology in combination with ADT. The ultimate goal of these trials would be to enhance the current efficacy of drugs used for treating advanced PCa.

## 2. Biology of the Androgen-AR Axis

**Figure 1:**
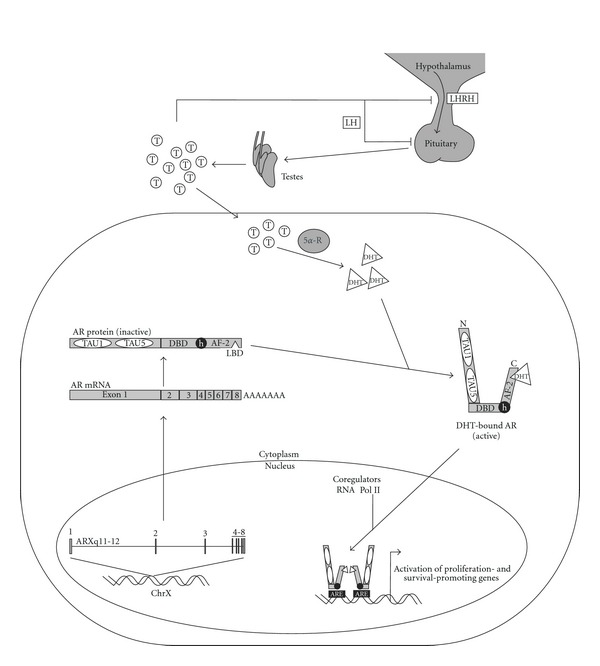
The androgen-AR axis. Testicular testosterone (T) production is regulated by the hypothalamic-pituitary axis of the endocrine system. In prostate cancer cells, T is rapidly converted to dihydrotestosterone (DHT) by 5 *α*-reductase (5*α*-R) enzyme activity. The AR gene is composed of 8 exons, with exon 1 encoding the N-terminal TAU1 and TAU5 transcriptional activation domains, exons 2 and 3 encoding the AR DBD, and exons 4–8 encoding the AR hinge (h) region and COOH-terminal LBD and AF-2 domains. DHT binding to mature AR protein induces nuclear translocation. Active AR binds target genes and recruits coregulatory proteins and components of the basal transcriptional machinery to achieve transcriptional activation. Abbreviations are defined in the text.

The androgen-AR axis regulates activity of the AR transcription factor, which is a master regulator of the prostate lineage. The lineage dependency hypothesis is an offshoot of the oncogene addiction hypothesis [10], stating that tumor progression requires the activity of master regulators that play key tissue development and/or survival roles [11]. In line with these criteria, AR signaling is an absolute requirement for the development and homeostasis of normal prostate tissue, and AR signaling is also an absolute requirement for the development and progression of PCa. The hypothalamic-pituitary axis stimulates testosterone production in by the testes (Figure 1). Circulating testosterone is bound by sex hormone binding globulin and albumin and only 1-2% exists in free, unbound form. This free testosterone diffuses into target cells of the prostate, testis, adrenal, skin, muscle, bone, and adipose tissue where it is irreversibly converted into a more potent biologically active metabolite, dihydrotestosterone (DHT) by action of 5 *α*-reductase in some, but not all, tissue types (depending on the presence of type I or II isoenzyme) [12]. Both DHT and testosterone exert their biological activities by binding to the AR, a 110 kDa member of the nuclear receptor superfamily of ligand-activated transcription factors. The AR gene is located on the X chromosome at position Xq11-12; therefore, males have a single genomic AR copy. The AR locus is approximately 180 kb in length, and modularity of the AR protein is reflected by the modular organization of exons within this locus (Figure 1). AR Exon 1 encodes the entire 538 amino acid AR NH2-terminal domain (NTD), exons 2 and 3 each encode one of the two zinc fingers constituting the 89 amino acid DNA binding domain (DBD), and exons 4–8 encode the 292 amino acid COOH-terminal domain (CTD) [13]. The AR NTD is structurally flexible [14, 15] and contains two transcriptional activation domains termed transactivation unit (TAU)-1 and TAU5. Additionally, transcriptional activation function-2 (AF-2) is a well-characterized transcriptional activation domain in the AR CTD that forms a shallow hydrophobic groove upon LBD binding to testosterone or dihydrotestosterone (DHT). Active AF-2 serves as a docking site for the NR-box motif of well-characterized AR coactivators such as SRC-1, -2 and -3 [16].

In the unliganded state, the AR resides primarily in the cytoplasmic compartment of prostate epithelial and stromal cells, in a stable complex bound to heat shock proteins (Hsps). DHT binding to the AR leads to higher AR stability, greater biological activity, and slower dissociation than testosterone. Upon ligand activation, changes occur in the composition and conformation of the AR-Hsp complex, leading to nuclear translocation [17]. In the nucleus, the AR binds as a dimer to androgen response elements (AREs) in promoter and enhancer regions of target genes such as prostate specific antigen (PSA) and leads to transcription of mRNAs (Figure 1). Tumor regression is induced either chemically with LHRH analogs or surgically by orchiectomy, which initially reduces the intracellular concentration of DHT [17] resulting in inhibited proliferation and increased apoptotic death of androgen-dependent PCa cells. 

## 3. Clinical Effects of ADT and Predictive Biomarkers

The clinical efficacy of ADT in advanced PCa is heterogeneous and ranges from a few weeks to several years, with an overall median time of 18 to 30 months [5–8]. Approximately 10% (range 6%–14%) of advanced PCa patients treated with ADT have an extremely short time to disease progression, lasting from a few weeks to months. To monitor response to ADT, serum PSA has been used extensively. Unfortunately serum PSA levels have not been demonstrated to have clinical validity with respect to predicting response to ADT [18–20], perhaps because PSA does not adequately reflect the heterogeneity in tumor biology observed in PCa. Beyond serum PSA, other clinical and tumor factors (such as Gleason score), have been evaluated as predictors of duration of ADT response [21, 22], but results have been inconclusive. The lack of predictive markers in this setting is a major unmet need in the management of advanced PCa. Indeed, identifying short treatment responses could allow the possibility of combining traditional ADT treatments with either more aggressive chemohormonal combinations or the use of emerging and novel drugs targeting the androgen-AR axis (abiraterone and TAK-700, CYP17A1 inhibitors that block androgen synthesis, or MDV3100, a next-generation antiandrogen) to enhance outcome [23] and prolong time to disease progression into a castrate-resistant state. Castrate resistant PCa (CRPC) is typically fatal over time, and while testosterone depletion remains an unchallenged standard for treating advanced stage hormone sensitive disease, “castration-recurrent” stages remain dependent and sensitive to further hormonal manipulations. Many molecular and cellular changes have been shown post “castration” to facilitate ongoing AR activity during ADT (reviewed in detail in [24–28]). As outlined in the following sections, many of these changes are due to somatic alterations of the AR gene, or components of the AR pathway, and therefore may have predictive value in the clinical setting. Similarly, nonsomatic inherited (germline) variation in an individual's hormone biosynthesis and metabolism pathway-associated genes may also contribute to the heterogeneity of response to castration and in the postcastration setting. At present, there is a keen and extensive ongoing scientific inquiry that focuses on both of these aspects.

## 4. Somatic Alterations in AR Pathway Genes Influencing AR Activity in PCa Cells 

Knowledge of somatic alterations in the PCa genome continues to expand at a rapid pace. Numerous early studies laid a broad foundation by assessing copy number gains and losses in tumor DNA using low-resolution comparative genomic hybridization (CGH) with spotted or manufactured arrays. A combined analysis of 41 published PCa CGH studies, which represented a total of 872 individual tumors, revealed multiple genomic regions that displayed frequent gain or loss in the PCa genome [29]. Subsequent reports employing high-resolution copy number analysis (>1 million probes) have provided detailed compendia of focal genomic gains and losses throughout the genomes of localized PCa as well as metastatic CRPC [30, 31]. Most importantly, by interrogating the genomic landscape in multiple metastases per patient, these and other studies have demonstrated that metastatic disease is monoclonal in nature and can be traced back to a single common origin [30, 32, 33]. More recently, exome sequencing has been performed in clinical specimens and PCa xenografts representing localized disease and CRPC, revealing the spectrum of somatic alterations in protein coding genes, and their putative role(s) in PCa progression [34–36]. Finally, whole-genome paired-end resequencing has been performed on 7 human primary PCas and their paired normal counterparts [37]. This study demonstrated that the mutation frequency in PCa is relatively low at 0.9 per megabase but also revealed a large number of copy-neutral rearrangements that would not be detected by traditional CGH approaches. In the case of PCa, these genomic studies are of particular clinical importance, because RNA expression profiles do not differentiate between low- and high-risk disease [36]. However, distinct PCa risk-groups emerge when copy number gains and losses are assessed [36]. Based on these important studies, it is anticipated that knowledge of the nucleotide sequence and chromosome structure of individual PCa genomes will lead to better treatment decisions and overall patient management when ADT has to be initiated.

These large-scale genomic studies have also reinforced the importance of the AR gene in PCa, particularly when the disease has progressed to a CRPC phenotype. For example, direct alterations in the AR do not occur in primary PCa but occur in 58% of metastatic cases. Integration of exome sequence, genome copy number, and gene expression data demonstrated that the broader “AR pathway” is altered in 56% of primary PCa and 100% of metastases. Therefore, direct alterations in the AR and broader alterations affecting the androgen-AR axis are among the most frequent events that occur in PCa development and progression. 

### 4.1. AR Gene Amplification and Copy Number Gain as Novel Predictive Biomarkers of ADT Response

One mechanism thought to increase AR protein expression is amplification of the AR gene. For example, an early study that analyzed the relationship between AR gene amplification and AR protein expression levels in matched androgen-dependent and CRPC tumors showed that 80% of tumors which acquired amplification of the AR gene also exhibited higher expression levels of AR protein [38]. Historically, AR gene amplification has been assessed in PCa cell lines and tissues using fluorescence *in situ* hybridization (FISH). These FISH-based studies have indicated that AR gene amplification occurs at a rate of 20–33% in CRPC [30, 38–41] but is rare in primary PCa [31, 38]. The absence of AR gene copy number alterations in primary PCa is corroborated by low- and high-resolution genomic data from multiple studies [29, 31, 36]. However, FISH may have provided an artificially low estimate of the prevalence of AR gene amplification in CRPC. For example, high-resolution copy number analysis of 58 CRPC metastases from 14 rapid autopsy subjects using the Affymetrix SNP6.0 platform identified increased AR gene copy number in 13/14 (93%) subjects [30]. However, high-level AR gene amplification (*AR* copy number > 4) was only observed in 5/14 (36%) subjects, which closely matches previous FISH-based estimates of AR amplification prevalence. This suggests that FISH may fail to identify AR amplicons that are focal in nature, which was demonstrated recently in a study that compared CGH-based AR gene copy number to AR FISH signals in cells derived from CRPC tissues [33]. Remarkably, this study had used fluorescence-activated cell sorting (FACS) to sort heterogeneous cell populations from CRPC biopsies based on DNA content. This allowed for the separation of aneuploid and diploid tumor cell populations and assessment of genomic aberrations that would have been obscured in mixed tumor cell populations. In one patient, multiple biopsies were obtained over an 8-year period at time points when the patient had displayed disease progression and switched AR-targeted therapies. This was particularly informative, because it demonstrated that cell populations with differing degrees of AR amplification existed within a single tumor and that these populations appeared to respond differently to orchiectomy versus bicalutamide [33]. This heterogeneity has also been observed in circulating tumor cells (CTCs). For example, one study that carried out AR FISH on CTCs from patients with CRPC found evidence of high-level AR amplification in 38% of samples analyzed [42]. However, a similar study of CTCs reported that 100% of patients with CRPC (*n* = 33) had evidence of AR amplification in at least one of their CTCs, with considerable heterogeneity between CTCs originating from a single patient [43]. Overall, these studies clearly demonstrate that AR gene amplification and/or copy number gain are frequent and important events during PCa progression. Changes in AR copy number are restricted to CRPC and do not appear to occur in primary PCa, indicating that these events are directly associated with the development of resistance to ADT. 

### 4.2. AR Gene Mutations as Predictive Biomarkers of ADT Response and Post-ADT Therapy

AR mutation is a mechanism of therapy resistance operating in a subset of CRPC. This area of PCa biology has been reviewed extensively [44, 45], and a database exists with an up-to-date compendium of AR mutations found in human disease [46]. The prevalence of AR mutations in CRPC found in different studies ranges from a low-end 10% rate to a high-end 100% rate [47–54]. Importantly, the study that identified AR mutations in 100% of CRPC specimens sequenced the equivalent of 20 separate full-length AR mRNAs per specimen, which would provide a sampling of multiple cell types and AR gene copies at this stage of the disease. This study also restricted analysis to CRPC specimens from patients that had been treated with bicalutamide and/or flutamide, which may provide stronger selective pressure for emergence of tumor cells harboring AR point mutations. Point mutations in the AR LBD have been interrogated extensively, and the majority (79%) appears to be confined to three discrete regions that make up only 8% of the AR coding sequence [55]. Functional study of several of these AR LBD mutations has provided a mechanistic explanation for resistance to ADT in some tumors. For example, mutations clustered within the AR signature sequence (amino acids 701–730) have been shown to alter the specificity of AR-ligand interactions, allowing inappropriate activation by alternative steroidal ligands such as adrenal androgens, glucocorticoids, and progesterone [56–58]. Mutations within a region adjacent to the AF-2 domain (amino acids 874–910) confer a similar property of altered ligand specificity on the AR [59]. One such mutation, T877A, exists in the LNCaP cell line, as well as human CRPC following antiandrogen therapy with hydroxyflutamide. T877A AR can be activated by adrenal androgens, estradiol, progesterone, as well as hydroxyflutamide and cyproterone acetate [60]. The final region of the AR exhibiting a clustering of mutations in PCa is between the DBD and LBD of the AR (amino acids 670–678). Functional studies of these AR mutants have demonstrated an overall enhanced transactivation in response to DHT, as well as other steroidal and nonsteroidal ligands [61]. Relatively fewer studies have assessed mutations in the AR NTD, but these have demonstrated that mutations in this region are recurrent and randomly distributed [50, 54, 62, 63]. Although there is substantially more functional data to illustrate how AR LBD mutations can support resistance to AR-targeted therapy, the mechanisms by which AR NTD mutations may drive resistance have begun to emerge. For example, an AR E231G mutant discovered in the transgenic adenocarcinoma of the prostate (TRAMP) model of PCa was shown to be oncogenic when expressed from a prostate-specific promoter [64]. An E255K mutation, which is adjacent to a region that interacts with the E3 ubiquitin ligase CHIP, was shown to enhance AR protein stability in the presence and absence of ligand [54]. Finally, a recurrent W435L mutation was identified in 6 individuals, which converts the AR WxxLF motif in the transcriptional activation unit (TAU)-5 domain to an LxxLF motif. This domain has been shown to be important for intramolecular interaction between the AR N- and C- terminal domains [65] and also for androgen-independent transcriptional activity [66]. Functional analysis of W435L AR demonstrated that this mutation strengthened the N-C interaction and enhanced AR transcriptional activity on certain promoters in a cell-line-dependent manner. Despite this wealth of functional knowledge, it is also important to note that many AR mutations that have been reported in clinical PCa do not appear to have any functional impact in a limited repertoire of assays [67]. Therefore, it remains a challenge to assess which treatment-dependent mutations in the AR gene may be *bona fide* drivers of resistance to AR-targeted therapy.

### 4.3. A Putative Role for AR Gene Rearrangements as Biomarkers of Response to Post-ADT Therapy

Altered splicing and synthesis of COOH-truncated, constitutively active AR variant proteins has emerged as an important mechanism of PCa resistance to AR-targeted therapy [68–72]. More recently, focal rearrangements in the AR gene have been linked to the splicing alterations that favor synthesis of these AR splice variants. The CRPC 22Rv1 cell line was derived from a CWR22 tumor xenograft that relapsed following castration. These cells express two AR-derived mRNA species that would only be expected to arise under a scenario where AR gene architecture has been altered. The full-length AR mRNA expressed in these cells contains a duplicate copy of AR exon 3, and the AR 1/2/3/2b mRNA contains an upstream exon 2b spliced *downstream* of exon 3 [68, 73]. Subsequent genomic copy number analysis and cloning of break-fusion junctions demonstrated that the 22Rv1 AR locus contains an intragenic 35 kb tandem duplication [73]. This duplicated genomic fragment harbors all of the cryptic exons that are alternatively spliced in this model, giving rise to various truncated, constitutively active AR variant species [73]. This represented the first report of AR gene rearrangements being linked to splicing alterations in CRPC. Subsequent gene structure analysis in additional models of CRPC confirmed this new concept by identifying an 8.5 kb deletion of AR exons 5, 6, and 7 at the 3′ end of the AR gene in the LuCaP 86.2 xenograft [74]. This xenograft model expresses high levels of the exon-skipped AR v567es variant, and deletion of these exons from the genome provides a strong rationale for this expression pattern [71]. Similarly, the first targeted paired-end resequencing of the entire 180 kb AR gene in the CWR-R1 led to the discovery of a 50 kb intragenic deletion within AR intron 1 [74]. Importantly, this deletion marked cells within the heterogeneous CWR-R1 cell population that had an androgen-independent growth phenotype and an enhanced capacity to synthesize the truncated AR-V7 or AR3 variant [74]. 

These types of focal, medium-scale rearrangements are not easily detected by low-resolution legacy techniques such as FISH or array CGH. However, high-resolution genome copy number analysis using platforms such as Affymetrix SNP6.0 would be expected to identify focal (<50 kb) copy number gains and/or losses when data are analyzed with an appropriate breakpoint-finding algorithm. Indeed, reanalysis of genomewide SNP6.0 data from metastatic CRPC and primary PCa demonstrated that complex patterns of copy number gain and/or loss occur along the length of the AR gene, exclusively in CRPC [73]. This array-based discovery was further corroborated by a targeted multiplex-ligation-dependent probe assay (MLPA) approach with DNA derived from CRPC cell lines, xenografts, and clinical specimens [74]. Interestingly, the complex AR gene architectures detected with these SNP6.0 and MLPA approaches frequently coincided with an overall increase in AR gene copy number, indicating that amplified AR “alleles” may harbor rearrangements, with the overall gain-of-function being a greater diversity of AR species expressed in CRPC cells in addition to increasing expression levels of wild-type AR. Indeed, the mouse AR-V4 (mAR-V4) isoform recently discovered in the Myc-CaP cell line was shown to arise from splicing of a cryptic exon-located 1 Mbp *upstream* from an amplified AR locus [72]. Thus far, rearrangement breakpoints have only been mapped and characterized in cell- and xenograft-based models of PCa progression [73, 74]. Therefore, further investigation is required to understand whether this new class of AR gene alterations plays a role in supporting resistance to AR-targeted therapies in clinical disease.

### 4.4. Somatic Alterations in Genes Involved in the Androgen-AR Axis

The earliest somatic alterations that occur in the androgen-AR pathway are fusions between 5′ genomic regulatory elements such as *TMPRSS2* and members of the Ets transcription factor family [75]. In this case, these fusions bring the expression of Ets transcription factors such as ERG or ETV1 under direct transcriptional control of the AR, effectively creating a completely new androgen-regulated gene and component of the androgen-AR axis. A decrease in ERG levels as a result of ADT-mediated AR repression may explain some of the growth-inhibitory effects of ADT in PCa [76]. Moreover, ERG expression in fusion-positive CRPC tumors reaches levels similar to untreated fusion-positive PCa, indicating that aberrant AR reactivation drives ERG reexpression when tumors develop resistance to ADT [76]. More recently, a novel gene fusion between the androgen-regulated *C15orf21* promoter and the Myc transcription factor was identified in a patient with aggressive PCa [77]. The *MYC* gene is frequently amplified in PCa [36], and it is conceivable that strong AR-mediated activity of the *C15orf21* promoter could drive Myc expression to a similar high level as Myc gene amplification.

In addition to gene fusions allowing Ets family members (and perhaps other factors such as Myc) to become new downstream components of the androgen-AR axis, traditional upstream regulators of AR transcriptional activity have also been shown to undergo somatic alterations in PCa cells. For example, the *HSD17B2* gene undergoes copy number loss, whereas the HSD17B3 gene undergoes copy number gain in CRPC specimens [78]. These genes encode the hydroxysteroid (17-beta) dehydrogenase-2 and -3 enzymes, which are responsible for catalyzing conversion between testosterone and the less active androgen, androstenedione [79]. Gain of *HSD17B3* and/or loss of *HSD17B2* would favor testosterone production in CRPC tissue, thereby increasing the level of AR activation. In addition, chromosome 8q13 displays broad copy number gain in 17% of PCa and amplification in 1.9% of primary PCa and 24.3–60% of metastatic CRPC [36, 78]. While these gains frequently include the entire q-arm of chromosome 8 [80], the 8q13 region harbors multiple genes including *NCOA2*, which encodes the AR coactivator SRC-2/TIF2. Mechanistically, *NCOA2* amplification was linked to increased expression of SRC-2/TIF2 mRNA levels [36]. This is important because multiple studies have documented SRC-2/TIF2 mRNA and protein overexpression in CRPC [16]. These studies have further demonstrated that increased protein levels of this coactivator can lead to AR hyperactivation and sensitive the AR to castrate levels of androgens [36, 81]. Remarkably, the *NCOA2* gene has also been shown to harbor somatic point mutations that are predicted to alter protein sequence in clinical PCa specimens [36]. Similarly, the AR coactivator p300 also harbors somatic point mutations in a subset of PCa [36]. Overexpression of p300 has been linked to androgen-independent activation of AR target genes by interleukin-6 [82, 83]. Additionally, the AR corepressor *NRIP1*/RIP140 and the AR corepressor *NCOR2*/SMRT have been shown to harbor somatic mutations in PCa [36]. While the precise effects of these mutations on AR activity, or the responsiveness of PCa cells to ADT have not been established, it is conceivable that multiple somatic alterations in the AR gene as well as its upstream regulators and downstream targets could have cumulative or synergistic effects that ultimately undermine efficacy of AR-targeted therapy.

## 5. Germline Variants in Androgen-AR Axis ****Genes and Response to ADT

The availability of information from the Human Genome Project has raised interest in investigating a role for host genome variation in predicting response to chemotherapeutic medications. A nascent, but very promising class of pharmacogenetic predictive markers are host (germline) genetic variations and their association with response and toxicity to chemotherapeutic agents; some successful examples include *TPMT* and thiopurine toxicity, *UGT1A1* and irinotecan toxicity, *CYP2D6* and tamoxifen efficacy, and *ERCC1* or *GSTP1*/*GSTP1*-I105V and oxaliplatin response and survival [84, 85]. In PCa, early studies have detected associations of single nucleotide polymorphisms (SNPs) from several genes involved in hormone metabolism with duration of response to ADT, including *CYP19A1*, *HSD3B1*, *HSD17B4 *[85], and *TRMT11* [86], as well as genes involved in androgen transport including *SLCO2B1* and *SLCO1B3* [87]. Interestingly, the effects of inherited variation on clinical outcomes of disease and treatments unlike somatic mutations in the tumor genome may depend on the geographic origin of patient populations. For example, germline variants in estrogen and androgen receptor binding sites in Han Chinese patient populations appear to affect clinical outcomes in advanced prostate cancer patients receiving ADT. In one study, variation in *BNC2* (rs16934641) affected disease progression for patients treated with ADT for hormone sensitive disease, while variation in another estrogen receptor binding site related gene *TACC2* (rs3763763) was associated with prostate cancer specific mortality, and variation in a third related gene *ALPK1* (rs2051778) along with *TACC2* (rs3763763) was associated with all-cause mortality in advanced stage disease [88]. Similarly, variation in androgen receptor-binding site genes *ARRDC3* (rs2939244); *FLT1* (rs9508016). *SKAP1* (rs6504145) in Han Chinese populations was detected to affect prostate cancer specific mortality in advanced prostate cancer populations, and variation in *FBXO32 *(rs7830622) and *FLT1* (rs9508016) appeared to affect all-cause mortality in advanced prostate cancer [89]. On the other hand thus far, no inherited variations in AR, estrogen receptor-1 (ESR1), or estrogen receptor-2 (ESR2) genes have been found associated with prostate cancer aggressiveness or with the efficacy of androgen deprivation in Caucasian populations [90]. These inherited variations in candidate genes/pathways are at a stage of development which still requires clinical and functional validation in independent patient populations and ultimately prospective validation in clinical trials. Nevertheless, these discoveries support a role for using predictive and prognostic markers based on host genetic variation in prostate cancer in the future.

## 6. Challenges of Obtaining Biomarkers of the ****Androgen-AR Axis in CRPC

The two broad challenges for achieving the long-term goal of personalizing treatments in advanced PCa stages included a current lack of an adequate and systematic prospective collection of cancer tissue and lack of availability of functional xenograft models from patient tumor samples. Meanwhile, the rapid advancement and availability of next-generation sequencing technologies has permitted quick and comprehensive characterization of genomic changes. Drug development and selection in oncology has thus focused on the identification of specific abnormalities in genes and gene pathways. These next-generation sequencing technologies could be particularly useful for analysis of the entire tumor genome for alterations in genes and gene pathways associated with response or resistance to standard hormonal and chemotherapy interventions but have yet to make a clinical impact. In the advanced PCa stage, a critical need exists for the identification of the heterogeneous somatic changes characterizing this stage that are “druggable,” as this disease stage is inevitably fatal. Identification, functional validation, and clinical application of pathways responsible for the malignant phenotype and drug response at this stage based on the genomic status of the patient's tumor are more likely to lead to promoting longevity and quality of life. However, this will involve high-throughput analyses of advanced PCa stage tumors via next-generation sequencing, gene expression (RNA-seq), and CpG methylation, followed by an integrated analysis of the molecular signatures associated with response to treatments. Assessing germline variants in androgen-AR axis genes is relatively straightforward because germline DNA can be obtained from blood. In contrast, assessing somatic alterations in androgen-AR axis genes is more challenging, because this requires direct sampling of tumor tissue or tumor-derived cells. An additional challenge is disease heterogeneity, particularly in the metastatic setting, because different tumor cell populations are likely to have different responses to certain forms of ADT. Therefore, to obtain a clear picture of the somatic alteration landscape, it would be very informative to sample various tumor cell populations prior to therapy and also assess which of these populations persist during/after therapy. In the immediate future, prior to developing a systematic and prospectively annotated clinical database of tumor samples obtained from this stage, one possible way to sample the overall tumor landscape is through CTC capture. 

CTC can provide a snapshot of the molecular composition of the cell populations driving the progression of the disease at a given time. Once identified, CTCs can be analyzed and can provide information that may direct therapy to appropriate targets in an individual patient at the time of treatment decision. Several molecular markers that may impact future therapies have been detected in CTCs, including *MYC* amplification, *TMPRSS2*-Ets gene fusions, *PTEN* deletion, Her-2/NEU, EGFR, and insulin-like growth factor [36, 42, 43, 91, 92]. 

CTC analysis is dependent on the detection technique used to identify them. CellSearch has been regarded as the standard technology, using magnetic separation based on epithelial cell adhesion molecule (EpCAM) antibody-coupled ferrofluid, and based on rigorous morphologic criteria and expression of cytokeratins, DAPI, and excluding CD45 staining [92, 93]. Newer assays, such as FACS-based methods, increase the sensitivity in detection of CTC. CTC recognized by FACS approaches has been shown to express prostate-specific mRNAs such as PSA, AR, and TMPRSS2, suggesting that the increased sensitivity does not come at a price of a decrease in specificity [93]. However, in general, genomic studies with CTCs are challenging because current platforms are designed for CTC enrichment as opposed to CTC purification. Therefore, captured cellular material consists of all cells that are positive for EpCAM. As a result, cytogenetic techniques such as FISH are the current standard for genomic analysis of CTCs, because this allows for specific interrogation of genomic anomalies in EpCAM-positive cells that meet the cytometric criteria of a CTC. Any DNA or RNA purified following CTC enrichment will be extremely heterogeneous, because it will be derived from all EpCAM-positive cells and not just CTCs. Nevertheless, AR mutations have been detected successfully by RT-PCR analysis of RNA purified following CTC enrichment, with 57% of individuals studied displaying at least 1 mutant form of the AR [94].

However promising as a source of molecular profiling, CTCs may not reflect the entire molecular milieu explaining the advance of CRPC. Stromal epithelial interactions and conditions of the host tissue may not be adequately assessed in CTC molecular profiling. Obtaining metastatic tissue samples provides a richer material for molecular analysis. Samples usually include stromal and epithelial elements and are a better reflection of the microenvironment that allows metastatic implants to grow and expand. However, availability of metastatic material is limited because few clinical indications exist today for sampling of metastatic lesions. Sampling requires invasive procedures associated with significant morbidity, usually consisting of needle sampling to obtain the tissue. This results in small-sized samples, frequently barely sufficient for histopathologic diagnosis, with limited if any amount available to collect frozen tissue. While buffered formalin compatible with molecular testing is becoming the standard fixative in most pathology laboratories, there is still large variability regarding time to and total time of fixation, which may impact certain molecular techniques. Furthermore, since the vast majority of metastatic PCa involves bone, sampled tissue frequently requires decalcification processes for proper evaluation. Routine decalcification involves treatment of tissue with formic acid, resulting in significant degradation of DNA, and hence rendering samples unsuited for molecular biological testing. De Jong et al. have recently described a protocol for molecular genetic testing of decalcified formalin-fixed, paraffin-embedded tissue, with promising results [95]. 

Another limitation of tissue sampling is the heterogeneity within a biopsy sample as well as the variable presence of stroma or normal tissue, which can be a confounding factor for genomic analysis. Depending on the ratio of tumoral versus nontumoral cells, results from molecular testing may yield impaired results. Particularly challenging is the presence of abundant blood, necrosis, and inflammatory reactions in the sample, which may overshadow the DNA from the tumor cells. Cell enrichment techniques such as microdissection and laser capture microdissection can improve the sample quality for molecular analysis but are labor intensive. 

It is also possible to separate distinct clonal cell populations within an individual biopsy, which may be useful for identifying the cell types that respond to specific therapeutic interventions. For example, a recent study used flow cytometry-based DNA content measures to collect diploid and aneuploid tumor cell populations from PCa biopsy specimens, followed by genomic analysis using array CGH [33]. In this study, three longitudinal samples were available from a single PCa patient that had undergone various forms of AR-targeted therapy over an 8-year period. Upon emergence of CRPC, the aneuploid and diploid tumor cell populations both displayed AR amplification but with dramatically different patterns. Remarkably, the aneuploid tumor cell population, which displayed the highest AR copy number, disappeared following treatment with bicalutamide [33]. This clearly demonstrates that clonal evolution occurs over time in response to therapy and that tumor cells with different patterns of somatic alterations in the AR pathway may display variable responses to different modes of ADT.

## 7. Predictive Biomarkers and ****Clinical Trial Design

Knowledge of predictive biomarkers for patients with CRPC may lead to identifying novel pathways of early failure, which may lead to new therapeutic targets. For example, more aggressive therapeutic strategies could be used for patients who are destined to have a limited period of clinical response to ADT. In contrast, patients with biomarkers associated with long-term responses to ADT could undergo intermittent rather than the usual continuous ADT exposure and avoid the many toxicities of chronic ADT administration, including osteoporosis, loss of sexual libido, increased risk of diabetes and coronary artery disease, and metabolic syndrome. Indeed, the efficacy of intermittent ADT is noninferior to continuous ADT [96] but is not widely used for treating PCa patients. Thus, a future clinical impact of predictive biomarkers of ADT response would include an informed and rational development of drugs and their combination with ADT for the treatment of advanced PCa. Ultimately, this should enhance clinical care by moving the field towards individualized medicine, with the advantage of improving response durations and limiting side effects.

We now present examples of using emerging predictive biomarkers related to the androgen-AR axis as the basis of stratifying the metastatic PCa patient population for biomarker-based clinical trials. In contrast to a prognostic biomarker that is associated with clinical outcome regardless of ADT, a predictive biomarker provides information about treatment effect of ADT [97]. The traditional paradigm for identifying biomarker as a correlative or translational component of research study is post hoc and thus may not sufficiently serve for the determination, validation, or application of a predictive biomarker for ADT. Instead, biomarker status must be incorporated into the design of a prospective clinical trial that deems to determine, validate, or apply a predictive biomarker for ADT. Many biomarker-based clinical trial designs have been proposed in recent years. Some of them specifically discussed the use of genomic signatures in developing novel therapies in clinical trials [98, 99]. We will discuss a few of these with regards to their potential application for determining, validating, or applying genotypic variation in androgen-AR axis genes (germline and somatic/tumor) as biomarkers of response to ADT in prospective clinical trials. The primary endpoint is defined as time from initiation of ADT to systematic progression or biochemical progression in terms of a series of PSA increases. 

### 7.1. Enrichment Design

**Figure 2:**
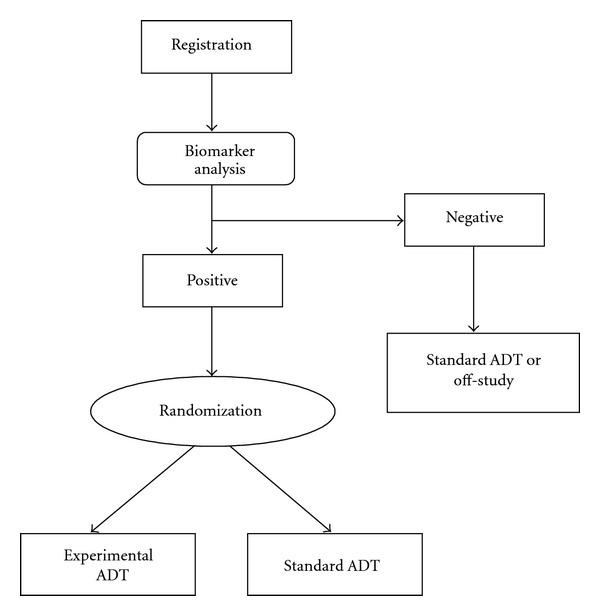
Enrichment design. Flow diagram of an enrichment clinical trial design for a predictive biomarker of ADT. Details are discussed in the text.

Enrichment design is suitable for established predictive biomarkers that are generally accepted with extensive evidence [100]. It requires screening all patients for their biomarker status before receiving ADT, but only the subset of patients with positive biomarker status, defined by a particular genomic feature that is deemed to benefit from ADT, will be randomized to receive control and experimental ADT. The rest of patients may either receive control therapy or be moved off-study (Figure 2). Two-stage adaptive accrual designs are further extensions to enrichment design in which interim analysis is used to determine whether to restrict or expand patient accrual to certain biomarker status. For example, the threshold sample-enrichment approach [101] enrolls only patients with positive biomarker and randomizes them into control and experimental ADT in the first stage and then expands to enroll all patients in the second stage if the interim analysis is statistically significant; an alternative approach [102] may enroll patients regardless of biomarker status and randomizes them into control and experimental ADT in the first stage and then restricts to enroll only patients with positive biomarker if the interim analysis is statistically insignificant.

### 7.2. Marker Strategy Design

**Figure 3:**
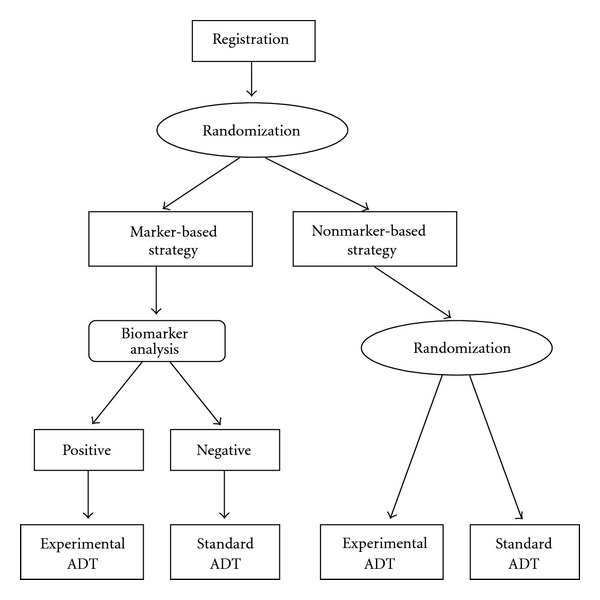
Marker strategy design. Flow diagram of a marker strategy clinical trial design for a predictive biomarker of ADT. Details are discussed in the text.

**Figure 4:**
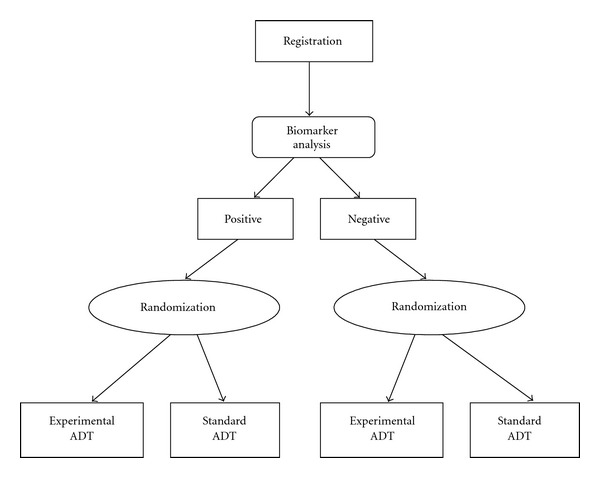
Marker stratified design. Flow diagram of a marker stratified clinical trial design for a predictive biomarker of ADT. Details are discussed in the text.

Marker strategy design and marker stratified design are both biomarker-based clinical trial designs that can be utilized to validate somatic mutations of AR or germline SNPs as predictive biomarker(s) of ADT. The marker strategy design [103] randomizes all patients for ADT into marker-based strategy cohort and nonmarker-based strategy cohort. For the marker-based strategy cohort, patients receive ADT based on their biomarker status, that is, patients with a positive biomarker receive experimental ADT and patients with negative biomarker receive control therapy (Figure 3). The deterministic assignment of treatment is based on the assumption that patients with a positive biomarker will be more likely to benefit from experimental ADT. For the nonbiomarker-based strategy cohort, patients will be randomized to receive control and experimental ADT. The marker stratified design [103] (also referred to as marker-by-treatment design) stratifies patients based on biomarker status and then randomizes patients into control and experimental ADT within each stratum (Figure 4).

### 7.3. Adaptive Signature Design

Adaptive signature design [104] unifies the development and validation of a predictive biomarker into a single two-stage clinical trial. While a genomic signature classifier can be generated from these somatic mutations of AR or germline SNPs in the first stage, it is prospectively used for stratifying patients into positive and negative biomarker status before randomizing them into control and experimental ADT in the second stage, similar to marker-stratified design. Unlike the previous design where only one biomarker is evaluated at a time, multiple germline or somatic variants in androgen-AR pathway genes can be considered through reduction into a single binary classifier for patient stratification.

### 7.4. Adaptive Randomization Design

Adaptive randomization designs [105, 106] are biomarker-based designs that intend to provide optimal treatment for patients. For example, germline or somatic variants in androgen-AR pathway genes could be incorporated into a regression model and response-adaptive randomization used to assign a current optimal treatment for each incoming patient based on their biomarker status. Moreover, more than two treatments of control and experimental ADT can be considered in these adaptive randomization designs. In this case, the predictive biomarker effect can be examined by testing the interaction between biomarker and treatment in the regression model.

## 8. Conclusion and Future Challenges

The recent advances in the medical management of advanced prostate cancer include successful translation and development of novel agents that target different components of the androgen-AR axis. This progress has taken considerable time considering that new agents for therapy of advanced prostate cancer still adhere to the basic principles established in the seminal work of Huggins and Hodges [2, 3]. Despite this progress it is clear that not all patients respond equally when treated either with initial standard ADT or with novel drugs targeting the androgen-AR axis. Underlying differences in individual tumor biology or inherited host variation or both may explain some of the variability in drug response and also may provide potential predictive biomarkers which can be clinically applied in the future. We propose that these trials should test novel combinations of standard ADT with a growing repertoire of drugs for the therapy of advanced prostate cancer, with the goal of enhancing treatment science in prostate cancer. A major hurdle at present is the paucity of clinically and functionally validated markers for final clinical testing using biomarker-based clinical trial designs. However, the potential of serum adrenal androgen levels as markers of response to oral ketoconazole [107] and intratumoral androgen levels as markers of response to abiraterone acetate [108] provide attractive illustrations of the potential value of using androgen axis components as predictive biomarkers of hormonal interventions. Ultimately, clinical implementation will require ongoing discoveries of potential markers, clinical and functional validation, and final clinical testing using biomarker-based clinical trial designs.
